# Subarachnoid Hemorrhage Causing Hydrocephalus: Narrative Review of a Neurosurgical Emergency

**DOI:** 10.7759/cureus.114559

**Published:** 2026-08-15

**Authors:** Nissar Shaikh, Fairoz Nadeem, Mansoor Nainthramveetil, Arshad Ali, Abdelnaser Thabet

**Affiliations:** 1 Surgical Intensive Care, Hamad Medical Corporation, Doha, QAT; 2 Anesthesia and Critical Care, Weill Cornell Medical College, Doha, QAT; 3 Anesthesia and Critical Care, Qatar University, Doha, QAT; 4 Anesthesiology and Critical Care, Hamad Medical Corporation, Doha, QAT; 5 Neurosurgery, Weill Cornell Medical College, Doha, QAT; 6 Neurosurgery, Hamad Medical Corporation, Doha, QAT

**Keywords:** acute & chronic, asah, csf, evd, hydrocephalus, lamina terminalis fenestration, lp shunt, motor and cognitive dysfunction, sah, vps shunt

## Abstract

Hydrocephalus is a common complication following aneurysmal subarachnoid hemorrhage (aSAH) and remains one of the most frequent causes of readmission in patients with subarachnoid hemorrhage (SAH). If left untreated, hydrocephalus may lead to significant neurological deterioration and can be fatal; therefore, it represents a neurosurgical emergency. This review summarizes the current understanding of hydrocephalus following SAH based on the available literature. Hydrocephalus occurs in approximately one third (1/3) of patients with SAH. Depending on the timing of onset, it can be classified as acute, subacute, or chronic hydrocephalus. The underlying pathophysiology is multifactorial and includes obstruction of cerebrospinal fluid (CSF) circulation by blood clots and blood degradation products, impaired CSF absorption due to inflammation and fibrosis, and in some cases increased CSF production.

Diagnosis is primarily based on clinical deterioration and neuroimaging findings, with computed tomography (CT) of the brain remaining the gold standard for detecting ventricular enlargement and confirming the presence of hydrocephalus.

Management of acute hydrocephalus mainly involves CSF diversion, most commonly through external ventricular drainage (EVD). In some patients, hydrocephalus may persist and progress to a chronic form requiring permanent CSF diversion with ventriculoperitoneal (VP) or lumboperitoneal (LP) shunts. Hydrocephalus following SAH is associated with increased mortality, prolonged hospitalization, and long-term neurological impairment, emphasizing the importance of early diagnosis and timely management to improve patient outcomes.

## Introduction and background

Cerebrospinal fluid (CSF) plays an important role in protecting and cushioning the brain and spinal cord [1]. Hydrocephalus occurs when there is an abnormal accumulation of CSF within the ventricular system of the brain, resulting in increased intracranial pressure, abnormal absorption due to blunting or obstruction by blood products, and sometimes subarachnoid hemorrhage (SAH) is also associated with intraventricular hemorrhage [2]. 

Hydrocephalus may occur as a result of congenital abnormalities or acquired conditions. In patients with aneurysmal subarachnoid hemorrhage (aSAH), hydrocephalus can lead to progressive neurological deterioration and, if not treated promptly, may result in fatal outcomes. Therefore, hydrocephalus following SAH is considered a neurosurgical emergency [1,2]. 

Hydrocephalus as a complication of aSAH was first described by Bagley in 1928 [1]. Later experimental studies demonstrated the development of hydrocephalus by injecting an iron-dextran mixture into the subarachnoid space of experimental animals [2]. The occurrence of hydrocephalus in non-aneurysmal SAH has not been extensively described; however, it has been reported to be more common in non-perimesencephalic SAH compared with perimesencephalic SAH [3]. In contrast, hydrocephalus is more frequently observed in aneurysmal SAH and occurs in approximately one out of six patients with aSAH [4]. 

Hydrocephalus is therefore considered a frequent but serious sequela of subarachnoid hemorrhage, as it can result in increased intracranial pressure and deterioration of neurological status. Following SAH, hydrocephalus most commonly develops due to obstruction of CSF pathways or impairment of CSF absorption, leading to ventricular enlargement and increased intracranial pressure [4]. 

## Review

Materials and methods 

A literature search was conducted using major databases, including Google Scholar, PubMed, the Cochrane Database, as well as conference presentations and abstracts. The keywords used for the search were "hydrocephalus in subarachnoid hemorrhage" and "hydrocephalus in aneurysmal SAH". Data were collected on various aspects of hydrocephalus associated with subarachnoid hemorrhage, including epidemiology, risk factors, classification, pathophysiology, diagnosis, and treatment. The above-mentioned keywords were searched for publications published between 1995 and 2025. The literature reviewed included prospective studies, retrospective studies, review articles, and case reports. Relevant information from these studies was collected, analyzed, and summarized in this review. 

Epidemiology 

Hydrocephalus is a frequent and serious complication of SAH. It occurs in up to 30% of patients with SAH, although the reported incidence varies widely in the literature, ranging from 6% to 67% [3,4]. 

The occurrence of hydrocephalus after SAH is closely related to the severity of the hemorrhage. Studies have reported that hydrocephalus develops in approximately 40% of patients with grade III SAH and 41% of patients with grade IV SAH [4]. 

Chronic hydrocephalus may develop in a subset of these patients and can lead to shunt-dependent hydrocephalus in approximately 6-37% of cases. Among these patients, around 8-10% require permanent shunt placement, which is often associated with longer hospital stays and increased healthcare utilization [5].

Classification

Hydrocephalus is defined as dilatation of the ventricles due to accumulation of CSF within the ventricular system. In SAH, hydrocephalus may occur due to obstruction of CSF circulation by blood or blood clots within the ventricles or the subarachnoid space. This form is referred to as obstructive or non-communicating hydrocephalus. 

**Figure 1:**
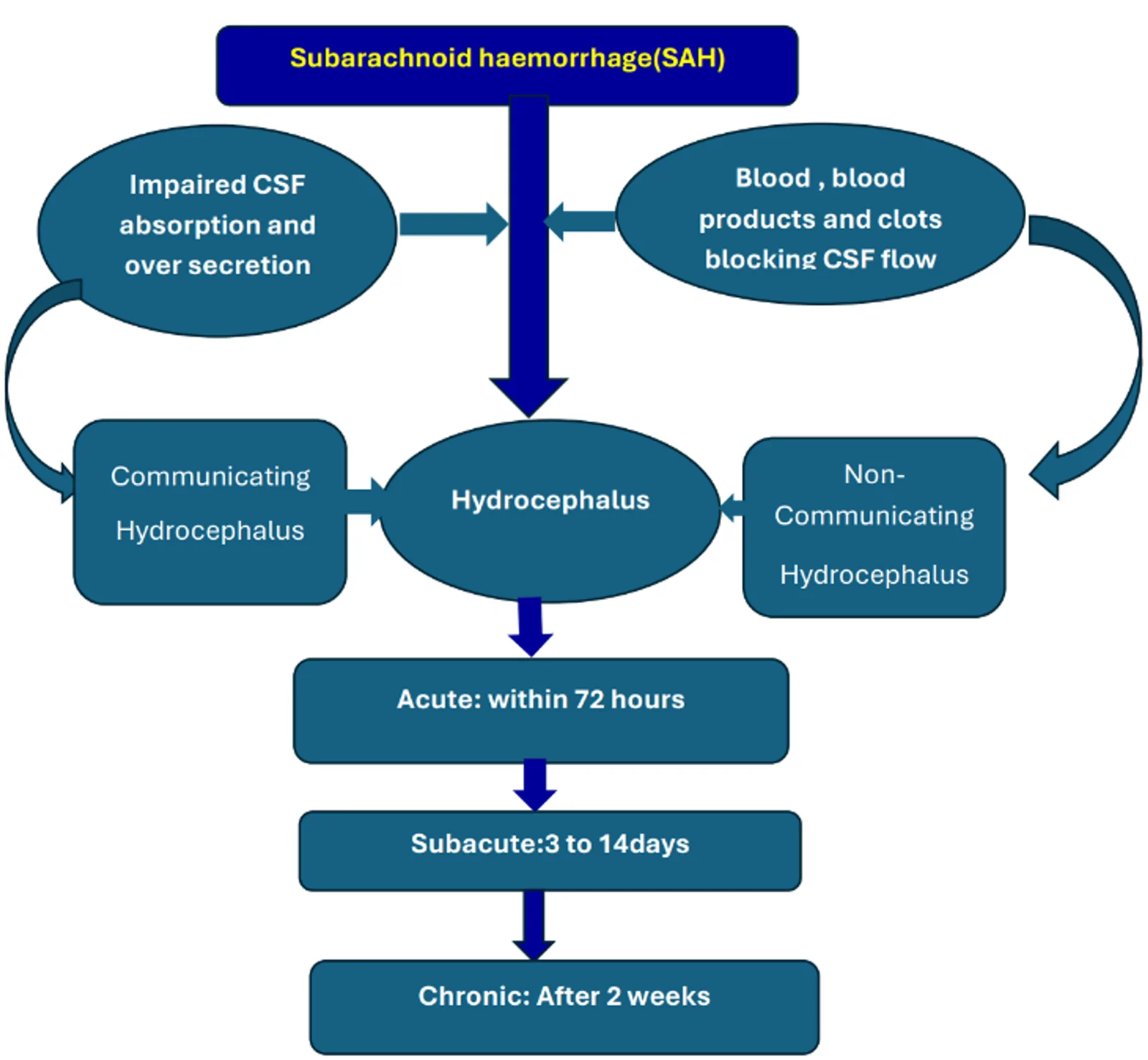
Types of hydrocephalus in SAH Original material produced by authors using Microsoft Word (Microsoft, Redmond, Washington). SAH - subarachnoid hemorrhage; CSF - cerebrospinal fluid

In SAH, blood may also interfere with the normal absorption of CSF at the level of the arachnoid granulations, resulting in communicating hydrocephalus (Figure 1). 

Hydrocephalus following SAH can also be classified according to the timing of onset. Acute hydrocephalus occurs within 72 hours after SAH and is mainly related to the presence of blood within the CSF pathways. Subacute hydrocephalus occurs between three and 14 days, usually due to inflammatory changes associated with blood clot breakdown that interfere with CSF circulation. Chronic hydrocephalus develops when ventricular enlargement persists or appears after two weeks, typically due to fibrosis and scarring within the subarachnoid space that impair CSF absorption (Figure 1) [6]. 

Risk factors

Hydrocephalus is a potentially life-threatening complication of subarachnoid hemorrhage (SAH). Early identification of risk factors is therefore important, as it allows clinicians to monitor high-risk patients closely and intervene early (Table 1). 

**Table 1:** Risk factors of hydrocephalus in patients with SAH SAH - subarachnoid hemorrhage; HCP - hydrocephalus; HTN - hypertension; EVD - external ventricular drainage; GCS - Glasgow Coma Scale; SDASH - Shunt Dependency in Aneurysmal Subarachnoid Hemorrhage; CHESS - Changes in Health, End-stage disease and Signs and Symptoms

Acute and subacute HCP	Chronic HCP
Patient age	All risk factors from the acute and subacute hydrocephalus plus
Known hypertensive or HTN, upon admission, post-operative HTN	Elderly patients
Low GCS	Female gender
Higher grade Intraventricular hemorrhage	Persistent inflammation
Rebleeding	Delayed EVD insertion
High amount of blood basal, anterior, lateral region brain	High Graeb score
Posterior circulation aneurysm	Aneurysm size
	Higher shunt dependency score SDASH
	Higher CHESS-Hübner SAH-VP score

The development of hydrocephalus after SAH is multifactorial and is mainly related to disturbances in cerebrospinal fluid (CSF) circulation. Intraventricular hemorrhage is one of the most important factors because blood within the ventricles can obstruct normal CSF pathways. SAH associated with posterior circulation aneurysms and hypertension at admission or postoperatively has also been linked to an increased risk of acute hydrocephalus [7]. 

Higher grades of SAH, indicated by Hunt and Hess or Fisher grading [8,9], along with rebleeding, are recognized risk factors for the development of hydrocephalus. A larger burden of blood within the ventricles or the subarachnoid space further increases the risk of acute obstructive hydrocephalus [10]. CSF production may increase in response to inflammation or infection, leading to excessive CSF accumulation and contributing to the development of hydrocephalus [11]. In up to 85% of patients with SAH, hydrocephalus resolves spontaneously within a few days. However, in some patients, persistent inflammatory processes, infection, or early fibrosis may impair CSF circulation and absorption. 

These mechanisms may lead to persistent or chronic hydrocephalus, which may require permanent CSF diversion using ventriculoperitoneal (VP) or lumboperitoneal (LP) shunts [11]. 

Additional factors associated with chronic hydrocephalus include advanced age, greater clot burden, elevated CSF protein levels, and repeated attempts at EVD weaning [12]. The Modified Graeb score, which quantifies intraventricular hemorrhage, has also been shown to predict the risk of chronic hydrocephalus [13]. Several predictive models, including the SDASH, CHESS, and SAH-VP scores, have been developed to estimate the likelihood of shunt dependency in patients with aneurysmal SAH [14-17]. 

Pathophysiology 

The pathophysiology of hydrocephalus following aneurysmal subarachnoid hemorrhage is complex and multifactorial. The presence of blood and its degradation products within the cerebrospinal fluid (CSF) disrupts normal CSF circulation and absorption. Blood clots may directly obstruct CSF pathways within the ventricular system, while blood products can impair absorption at the level of the arachnoid granulations and trigger inflammatory responses [10]. 

**Figure 2:**
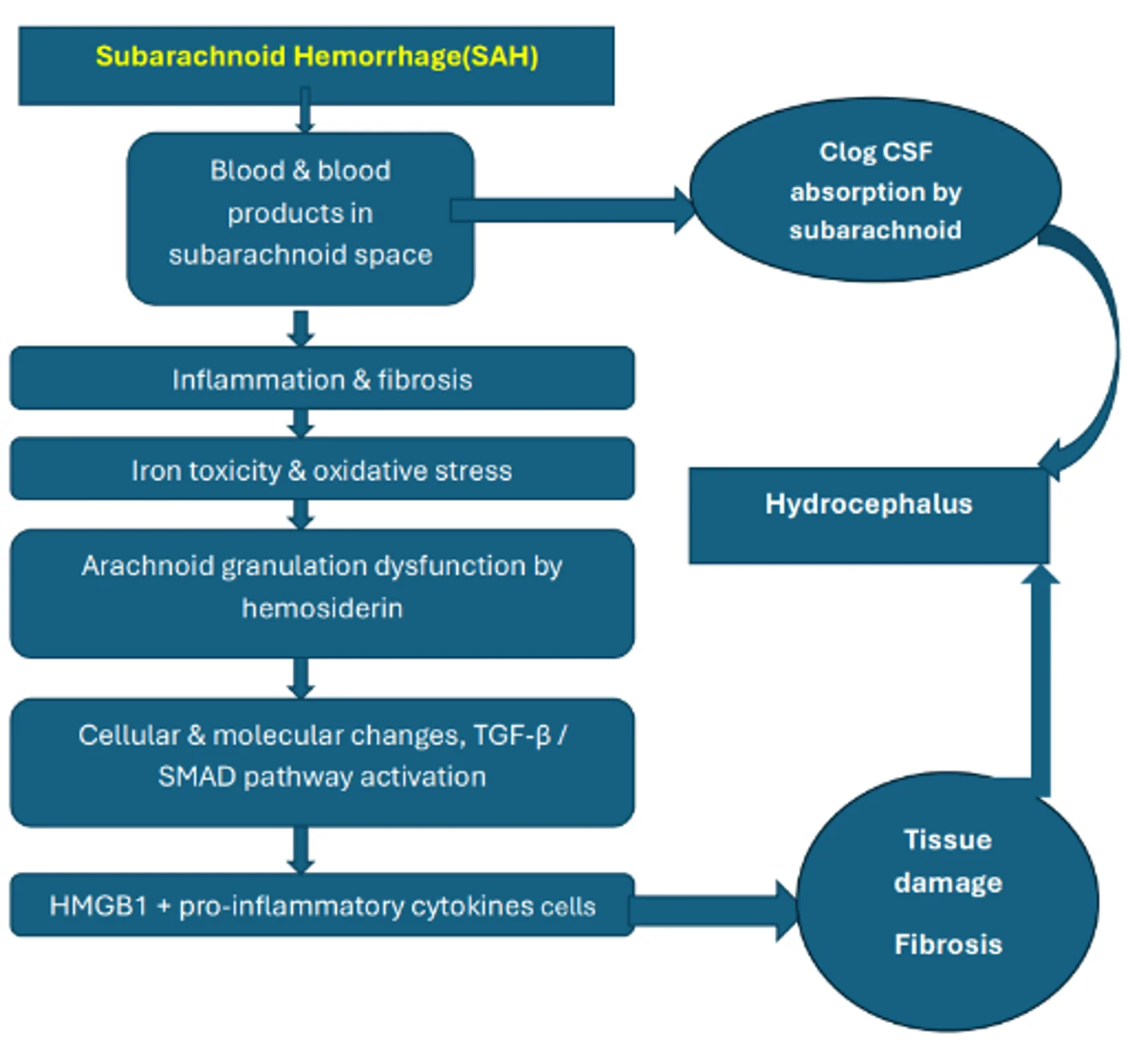
Pathophysiology of hydrocephalus in subarachnoid hemorrhage Original material produced by authors using Microsoft Word (Microsoft, Redmond, Washington) SAH - subarachnoid hemorrhage; CSF - cerebrospinal fluid

The development of hydrocephalus after aSAH can be explained by several mechanisms (Figure 2). 

First, obstruction of CSF circulation may occur when blood clots or degradation products such as hemosiderin accumulate within the subarachnoid space or ventricular system. These substances can block the arachnoid villi and granulations, reducing CSF absorption and clearance. In addition, inflammation associated with aSAH can lead to fibrosis and adhesions within the subarachnoid space and around the ventricles, further disrupting CSF circulation [18,19]. 

Second, aSAH alters normal CSF dynamics. Inflammatory reactions involving the choroid plexus may increase CSF production while CSF absorption is simultaneously reduced. This imbalance between CSF production and absorption results in ventricular enlargement and the development of acute or chronic hydrocephalus. 

Third, blood degradation products such as hemoglobin and iron compounds trigger significant inflammatory responses. These processes involve pro-inflammatory cytokines including interleukin-1 (IL-1) and tumor necrosis factor-α (TNF-α), as well as high mobility group box-1 (HMGB1) proteins. These inflammatory mediators contribute to disruption of the blood-brain barrier and promote fibrosis within the subarachnoid space [20]. 

Activation of the transforming growth factor-β (TGF-β) / SMAD signaling pathway further contributes to excessive extracellular matrix production and scarring within the CSF pathways. This process ultimately impairs CSF circulation and absorption, leading to the development of hydrocephalus [6,20]. 

Diagnosis 

Early diagnosis of hydrocephalus is essential, as prompt treatment can significantly improve clinical outcomes. In patients with SAH, diagnosis is primarily based on clinical deterioration, particularly a decline in the level of consciousness. Patients may present with sudden worsening of the Glasgow Coma Scale (GCS) score [21], accompanied by worsening headache, dizziness, vomiting, and papilledema. 

Neuroimaging plays a crucial role in confirming the diagnosis. Imaging studies are rapid and reliable for detecting hydrocephalus and other intracranial pathologies such as hemorrhage or ischemia in patients with aSAH. Computed tomography (CT) of the brain is the most used imaging modality and remains the gold standard for diagnosing hydrocephalus. 

On CT imaging, hydrocephalus is characterized by dilatation of the ventricular system. Enlargement of the temporal horns greater than 2 mm and a frontal horn width to internal skull diameter ratio greater than 0.5 are commonly used indicators of ventricular enlargement. 

Several radiological indices are used to assist in the diagnosis of hydrocephalus. The Evans index (EI) is defined as the ratio between the maximum width of the frontal horns of the lateral ventricles and the internal diameter of the skull at the same level [22]. An Evans index greater than 0.3 generally indicates ventricular enlargement and suggests hydrocephalus. 

Another useful measurement is the Bicaudate index (BI), which represents the ratio between the distance across the caudate nuclei and the width of the brain at the same level, adjusted for age [23]. An elevated BI also supports the diagnosis of hydrocephalus [24]. 

Magnetic resonance imaging (MRI) provides more detailed information about CSF flow dynamics and surrounding brain structures, although CT remains the preferred initial imaging modality because of its speed and availability. 

Additional diagnostic tools, such as intracranial pressure monitoring and fundoscopic examination, may also assist in identifying increased intracranial pressure associated with hydrocephalus. However, CT imaging remains the primary and most practical diagnostic modality in patients with SAH and suspected hydrocephalus [25]. 

Treatment 

The management of hydrocephalus following aneurysmal subarachnoid hemorrhage primarily focuses on removal of excess cerebrospinal fluid (CSF) and reduction of intracranial pressure. In the acute phase, this is usually achieved through CSF diversion techniques, most commonly external ventricular drainage (EVD). In selected cases, lumbar drainage or lamina terminalis fenestration during aneurysm clipping surgery may also be considered. 

EVD is the most frequently used treatment for acute hydrocephalus, particularly in patients with significant intraventricular hemorrhage. In addition to draining CSF and relieving intracranial pressure, EVD also allows continuous monitoring of intracranial pressure (ICP). During the acute stage, EVD helps remove blood and blood products from the ventricular system. Once CSF circulation stabilizes and ventricular size improves, the drain can be gradually weaned and removed (Table 2). 

**Table 2:** Comparison of shunt and fenestration therapies CSF - cerebrospinal fluid; VPS - ventriculoperitoneal shunt

Availability	Widely available; most commonly used procedure	Used in selected patients when VPS is unsuitable	Performed during aneurysm clipping surgery
Mechanism	CSF diversion from ventricles to peritoneal cavity	Lumbar CSF diversion to peritoneal cavity	Surgical opening of lamina terminalis to improve CSF circulation
Indication	Communicating and obstructive hydrocephalus	Mainly communicating hydrocephalus	Selected surgical cases during aneurysm repair
Advantages	Effective long-term CSF diversion	Avoids ventricular catheter placement	No implanted catheter or shunt device
Major complications	Infection, shunt malfunction, over-drainage, subdural hematoma	Infection, catheter obstruction, CSF leakage	It can get blocked by frontal lobe swelling or by clot easily, CSF leak, seizures

However, EVD placement is associated with several challenges. Blood clots may obstruct the catheter, and clearance of intraventricular blood may be slow. In addition, different strategies are used in clinical practice for EVD management and removal. Some centers prefer intermittent CSF drainage, while others maintain continuous drainage in symptomatic patients to promote clearance of blood and reduce catheter obstruction. Randomized studies have suggested that intermittent drainage may be associated with a lower risk of catheter blockage [26]. 

One of the major complications of EVD is infection, with reported rates ranging from 5% to 30%. The risk of infection increases with prolonged catheter duration, frequent CSF sampling, intensive care unit placement, and the use of non-antibiotic-impregnated catheters. The implementation of standardized EVD care bundles has significantly reduced infection rates, with some studies reporting infection-free rates of up to 99% [27]. 

There is also variation in the strategy used for EVD removal. Some clinicians prefer early removal once the drain is no longer required, which may reduce the risk of infection and shorten intensive care unit stay. Others prefer gradual weaning, in which the drainage level is progressively raised before the catheter is removed [28]. 

Lumbar drainage may be considered in selected patients with less severe hydrocephalus or as an alternative to ventricular drainage. Although lumbar drainage is generally considered safe, it carries potential risks including infection and hemorrhage [29-31]. 

Another surgical option is lamina terminalis fenestration, which may be performed during aneurysm clipping surgery to improve CSF circulation. Although this technique may facilitate CSF flow intraoperatively, recent studies suggest that it does not significantly reduce the long-term requirement for ventriculoperitoneal shunting [32]. 

In patients who develop chronic hydrocephalus, permanent CSF diversion is often required. The most commonly used procedure is ventriculoperitoneal shunt (VPS) placement, which provides continuous CSF drainage from the ventricles to the peritoneal cavity. Although VPS is an effective treatment, complications such as shunt malfunction and infection remain important clinical challenges [33,34]. 

Lumboperitoneal shunts (LPS) may be considered in patients who are not suitable candidates for VPS. Compared with VPS, LPS may have fewer complications related to ventricular catheter placement. However, VPS remains the preferred and more widely used treatment option in most patients with chronic hydrocephalus [35]. 

Recent therapeutic developments have focused on minimally invasive approaches to CSF diversion and strategies aimed at reducing inflammation and CSF overproduction. Emerging techniques include endovascular CSF shunting systems (e-shunt) and endoscopic third ventriculostomy (ETV). Ongoing research is also exploring therapies targeting inflammatory pathways to reduce fibrosis and improve CSF circulation following SAH [36] (Table 2). 

Prognosis 

Hydrocephalus significantly increases both morbidity and mortality in patients with subarachnoid hemorrhage. It remains one of the most common causes of hospital readmission following SAH [37-39]. 

Reported mortality rates associated with hydrocephalus after SAH vary widely, ranging from 3.6% to 46.7%, and may reach up to 50% in severe cases. In many patients, acute or subacute hydrocephalus may resolve spontaneously or improve following temporary CSF diversion (Table 3). 

**Table 3:** Newer therapeutic and research direction for treatment of HCP HCP - hydrocephalus; CSF - cerebrospinal fluid; ETV - endoscopic third ventriculostomy; SAH - subarachnoid hemorrhage; EVD - external ventricular drainage

Research area	Examples
Endovascular / minimally invasive CSF diversion	Endovascular CSF shunt systems; refined ETV
Modification of CSF flow and clearance	Intraventricular fibrinolysis for clot lysis; improved shunt systems
Anti‑inflammatory strategies	Anti‑inflammatory drugs, antioxidants targeting post‑SAH inflammation
Regenerative and biological therapies	Stem‑cell derived exosomes and neuroprotective therapies
Optimization of shunt management	Improved timing of EVD removal and transition to permanent shunt placement

Overall, the mortality rate associated with acute hydrocephalus has been reported to be approximately 28%. Among these patients, approximately 6-37% eventually develop chronic hydrocephalus and require permanent CSF diversion through ventriculoperitoneal or lumboperitoneal shunts. 

The presence of hydrocephalus in patients with SAH is associated with a higher risk of poor neurological outcomes. Patients with intraventricular or intracerebral hemorrhage have been reported to have a two- to threefold increased risk of death compared with those without these complications [40,41]. 

In addition to mortality, hydrocephalus contributes to substantial long-term morbidity. Survivors may develop cognitive impairment, motor dysfunction, and coordination difficulties, which can significantly affect quality of life. Hydrocephalus is also associated with prolonged intensive care unit and hospital stays, leading to increased healthcare costs. 

Approximately 10% of shunts may fail or require revision, resulting in additional hospital admissions and further treatment costs. Long-term outcomes vary considerably. Studies report that one-year survival among patients with shunt-dependent hydrocephalus is approximately 57%, decreasing to around 37% at five years. Furthermore, nearly 30% of survivors experience persistent neurological or cognitive deficits [42,43].

## Conclusions

Hydrocephalus is a common and potentially life-threatening complication of subarachnoid hemorrhage. It develops primarily due to the presence of blood clots and blood degradation products within the cerebrospinal fluid, which obstruct CSF circulation, impair CSF absorption, and trigger inflammatory processes that may lead to fibrosis. These mechanisms disrupt normal CSF dynamics and result in ventricular enlargement. Hydrocephalus may be classified as communicating or non-communicating, and according to the timing of onset, it may be present as acute, subacute, or chronic. Early diagnosis is essential, and computed tomography of the brain remains the gold standard imaging modality for detecting ventricular enlargement and confirming the diagnosis. Management depends on the stage and severity of hydrocephalus. In the acute or subacute phase, treatment primarily involves CSF diversion using external ventricular drainage or lumbar drainage to relieve intracranial pressure and restore CSF circulation. Patients who develop persistent or chronic hydrocephalus often require permanent CSF diversion through ventriculoperitoneal or lumboperitoneal shunts. Early recognition and timely management of hydrocephalus are critical for reducing mortality, neurological complications, and long-term cognitive impairment in patients with subarachnoid hemorrhage. 
